# A study on safety evaluation system of cultured foods among alternative proteins

**DOI:** 10.1007/s10068-024-01720-y

**Published:** 2024-09-30

**Authors:** Sojeong Heo, Gawon Lee, Do-Won Jeong

**Affiliations:** Department of Food and Nutrition, Dongduk Women’s University, Seoul, 02748 Republic of Korea

**Keywords:** Cultured meat, Novel food, New technology, Safety assessment

## Abstract

Food produced by cell culture has been approved in Singapore and the United States. Food safety evaluation systems of Singapore, the United States, Europe, and Korea were reviewed for producing cultured foods with new technologies. In Singapore, Europe, and Korea, safety evaluation of cultured meat is conducted by applying for such evaluation in the Novel Food system. In contrast, in the case of the United States, safety evaluation for cultured meat is conducted by the FDA and the USDA, considering it as an altered product of production methods, not a novel food. Required safety assessment data vary depending on whether the cultured meat is a novel food or a different food with a different production method. Accordingly, the current study presents differences between required documents according to the two distinct perspectives.

## Introduction

Cultivated meat, also known as lab-grown meat, is one of the artificially produced alternative proteins utilizing stem cell technology (Lee et al., 2020; Rubio et al., 2020). Alternative proteins contain animal proteins obtained from the livestock industry. They can be largely divided into cultured proteins cultured from plants, microorganisms, and animal cells (Chriki et al., 2022; Rubio et al., 2020). Interest in alternative proteins has increased as a response to the growing demand for animal proteins, public health concerns associated with current animal farming practices, and environmental and climate impacts (Jiang et al., 2020; Parlasca and Qaim, 2022; van der Weele et al., 2019). One of the most prominent examples of alternative proteins derived from plants is soy-based protein (Coleman et al., 2021). Unlike Europe, Asia has been consuming fermented foods using soybeans for a long time (Yang et al., 2011). As a result, protein-rich foods using soybeans are being consumed. However, countries that do not consume a lot of soybeans, such as those in Europe that typically eat food obtained from cattle farming, are unfamiliar with soybean patties that do not lead to satisfaction as a substitute of meat (Vural et al., 2023). An example of efforts to impart meat flavor to plant-based proteins is the addition of soy leghemoglobin (Froggatt and Wellesley, 2019; Shao et al., 2022). Although the consumption of plant-based proteins may seem unfamiliar in countries with a livestock-based diet, the long history of plant-based protein consumption in Asia contributes to the perception of safety (Rubio et al., 2020). However, questions remain as to whether animal-derived proteins such as cells cultured in a laboratory from animal cells using stem cell technology can be considered as safe as livestock animals. If they are equivalent to existing meat, they can be considered safe with an intake history of existing meat. However, if they are not, safety evaluation should be performed because they are novel foods with new technologies applied. Therefore, in this paper, we tried to deal with the equivalence and safety of animal-derived cultured foods.

## Are animal-derived cultured foods traditional meat or new?

Animal-derived cultured foods are referred to by various terms such as cultured meat, alternative protein, cell-cultured meat, alternative food, vegan meat, meat analogue, and meat alternative (Arora et al., 2023; Froggatt and Wellesley, 2019; Rubio et al., 2020). The term cultured meat is commonly used. It evokes traditional meat, which is why its usage is viewed negatively by livestock farmers (Chriki et al., 2022). Similarly, groups producing cultured meat are unfavorable to using terms such as analogue.

In Korea, it is defined as cell-cultured food ingredients (MFDS, 2024), while Singapore refers to it as alternative protein, using the term cultured (Cohen et al., 2021; Singapore Food Agency, 2024; Smith et al., 2022). In the European Novel Food Category, it is defined as cell or tissue cultures derived from animals/plants/fungi/algae (European Parliament and Council of the European Union (EU) 2015/2283, 2015). In the United States, it was initially termed cell-cultured meat. However, it is now referred to as cultured animal cells.

The reason for the mix of terminology is the emergence of a new type of food due to technological advancements and its animal origin. Thus, scientific comparisons are needed to determine if animal-derived cultured foods are equivalent to traditional meat (Lee et al., 2022; Schneider, 2013). Suggestions include comparing texture, structure, post-metabolism, color, and nutritional composition (Fraeye et al., 2020). However, comparative analysis of nutritional compositions of animal-derived cultured foods and traditional meat has not been presented yet (Broucke et al., 2023). Therefore, there is currently no scientific evidence to support the similarity between animal-derived cultured foods and traditional animal products. In other words, nutritional analysis alone is insufficient. Further investigations into the genome, transcriptome, metabolome, and proteome equivalence between animal-derived cultured foods and traditional meats are necessary.

## Safety evaluation system for animal-derived culture foods

If animal-derived cultured foods, as suggested above, demonstrate nutritional equivalence along with genome, transcriptome, metabolome, and proteome equivalence compared to traditional meat, they can be considered safe based on the consumption history of traditional meat. However, if they are not equivalent, it means they are new food ingredients in Korea and Novel Food in Europe due to application of new technology. Safety is evaluated through safety assessment systems required by each regulatory system.

### Safety evaluation system in Singapore

**Fig. 1:**
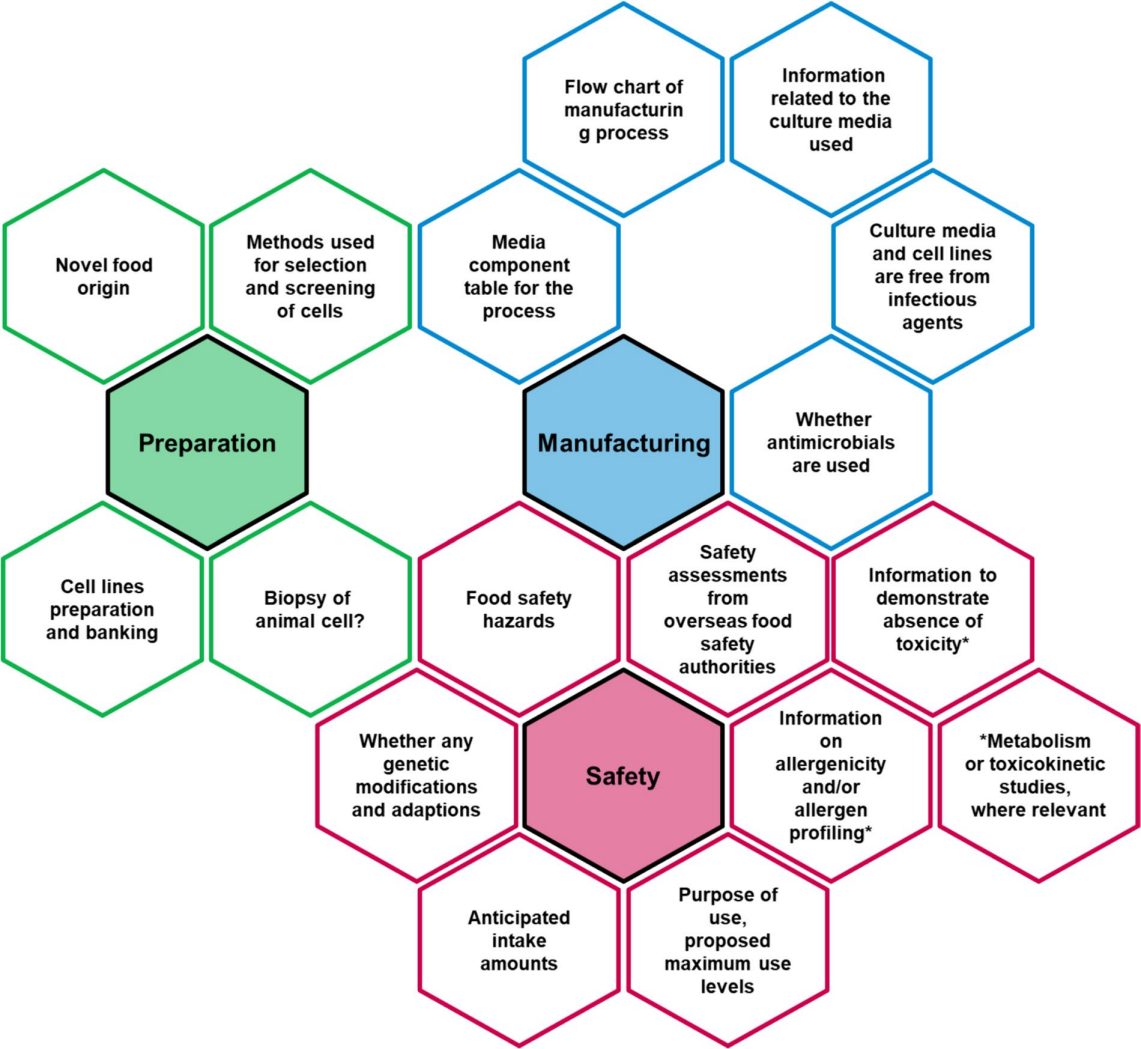
Checklist for cell-based food companies for Novel Foods in Singapore Food Agency

Singapore became the first country worldwide to approve the sale of cultured meat in December 2020. The Singapore Food Agency (SFA) is responsible for safety assessment of cultured foods. The SFA states that it evaluates the safety of cultured foods ranging from safety of cell lines used as the origin of cultured food to pre-production safety during the production process and safety of the end product. These contents are data that add more safety parts for cell lines to the safety evaluation of Novel Food. Cultured foods fall under the category of Novel Food. To obtain certification as a new food, applicants must submit to the SFA a self-assessment checklist of results related to potential food safety risks such as toxicity, allergenicity, safety of production methods, and dietary exposure resulting from consumption of Novel Foods (Singapore Food Agency, 2023) (Fig. 1 and Table 1). However, since cultured foods are produced using cells, additional information must be provided regarding cell origin, cell safety, storage and maintenance of cells, and risks of residual substances (such as media and antibiotics) used in cell culture (Ketelings et al., 2021; Lee et al., 2024). General components, toxicity, and consumption history are commonly evaluated for novel foods.

**Table 1:** The safety evaluation systems for cultured meat in Singapore, Europe, United States, and Korea

	Singapore	Europe	United States	Korea
Food Safety Regulatory Agency	Singapore Food Agency	European Food Safety Authority	Food and Drug Administration and U.S. Department of Agriculture	Ministry of Food and Drug Safety
Food Categroy	Novel Food	Novel Food	GRAS notification system	New food ingredients
Approval processes	1. Submit their applications 2. Fill in the Self-assessment checklist by the applicant 3. Complete an evaluation of a novel food	1. Submitted applications for Novel Food 2. Suitability checks by the EFSA 3. Validity check by the European Commission (EC) 4. Risk assessment 5. Publishes the scientific output by EFSA 6. Risk management to derive conclusions	1. GRAS review 2. GRAS by scientific procedures 3. Scientific data review 4. Experts agree to safety-in-use 5. GRAS	1. Applicant receives 2. Review by the Ministry of Food and Drug Safety 3. Approval of new food ingredients
Required information	- General components - Production process - Information related to cell lines used and scaffolding material - Toxicity and allergy - Safety of production methods - Dietary exposure - Risk assessments on any chemicals	- Production process - Product compositions, consumption history - Proposed uses and use levels - Anticipated intake - Metabolic effects through consumption - Nutritional information - Toxicity and allergy information	- Name of the GRAS material - Method of use - Detailed safety evaluation	- Cell donor and cell information - Development history, domestic and international recognition, and Consumption history - Manufacturing method - Characteristics of the food ingredient - Toxicological test data and intake evaluation data
Reference	(Singapore Food Agency, 2023; Lee et al., 2024)	(Regulation (EU) No 2015/2283; EFSA, 2021)	(Heo et al., 2024)	(MFDS, 2024)

### Novel food system in EU

**Fig. 2:**
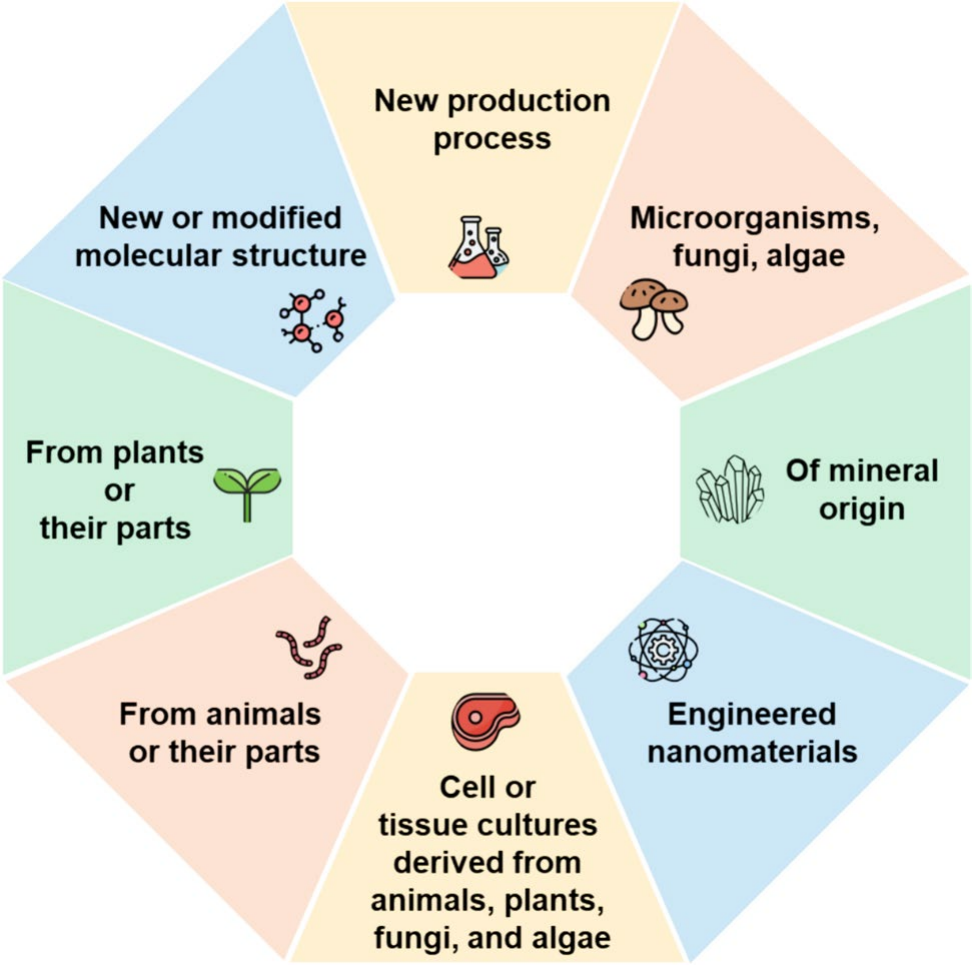
Novel Food categories according to (EU) 2015/2283 in Europe

In 1997, the European Union (EU) introduced the Novel Food system along with the (EC) No 258/97 regulation to establish a safety assessment system for food that has not been significantly used for human consumption purposes in EU. This regulation was then improved. In 2015, the EU adopted the Novel Food Regulation (EU) 2015/2283 to enhance the safety assessment system. The revised regulation aimed to clarify the definition of Novel Food, centralize administrative procedures, transition from individual recognition to notification system, and reduce the role of EU member states (EFSA, 2015). Particularly, the revised regulation defined Novel Food into eight categories (Fig. 2). One of these eight categories is food derived, isolated, or produced from cell culture or tissue culture originating from animals, plants, microorganisms, fungi, or algae, which includes cultured meat (European Parliament and Council of the European Union, (EU) 2015/2283, 2015).

As of now, no cultured meat has been approved as Novel Food. The European Food Safety Authority (EFSA) receives applications for Novel Food. After a validity check by the European Commission (EC), suitability checks by the EFSA are conducted within 30 days. In Europe, the approval process is strictly managed by the central system. This involves verifying if all documents submitted by the applicant are suitable. Subsequently, a risk assessment is carried out. After approximately nine months of risk assessment, the EC proceeds with risk management to derive conclusions (Table 1). Applicants are required to provide information on the manufacturing process of the novel food, product compositions, consumption history, estimated intake, metabolic effects through consumption (including absorption, distribution, metabolism, and excretion), nutritional information, toxicity information, allergy information, and more (EFSA, 2021). However, a senior scientific officer at the EFSA announced that no applications for cell-cultured food had been received up to 2023. Consequently, although the category for cultured meat has been established, there have been no safety assessments conducted to date.

**Fig. 3:**
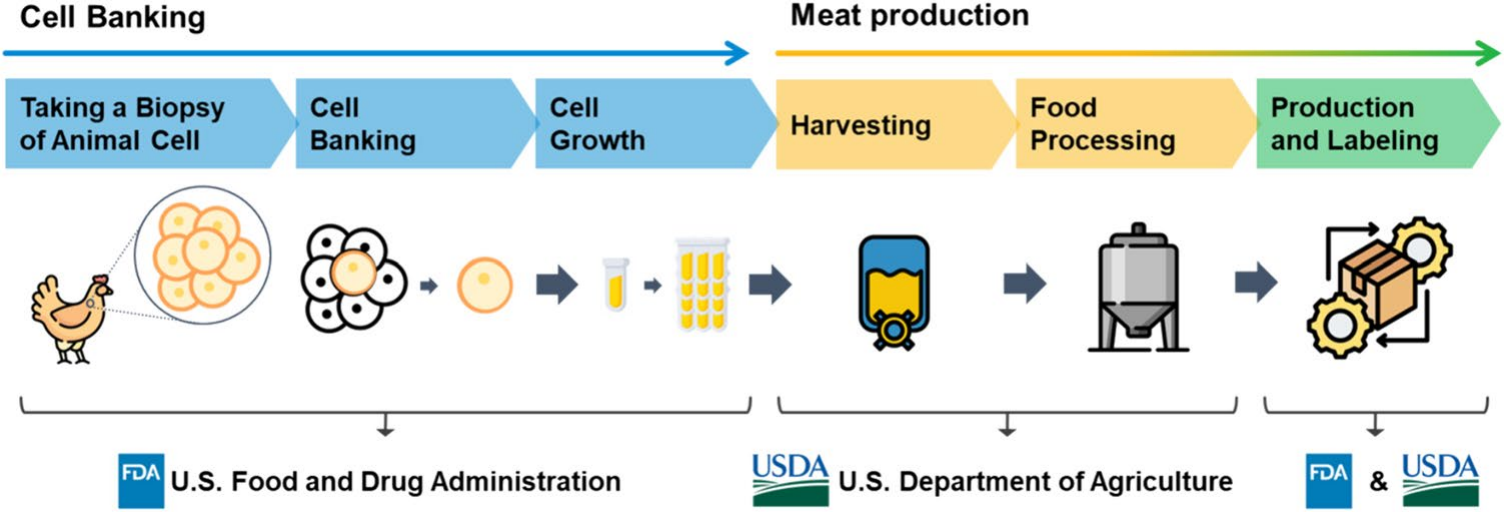
Overview of cultured poultry meat manufacturing process in USA

### Safety evaluation system in United States

In the United States, novel food ingredients have obtained Generally Recognized as Safe (GRAS) approval under the GRAS notification system. In the case of GRAS notification, if there is a history of consumption before 1958, before the GRAS system was implemented, it can be used as GRAS without a safety evaluation, but in other cases, the applicant can conduct a safety evaluation according to the approval procedure and then petition the FDA (Table 1). However, for cultured meat produced using new technology, safety evaluation is approached through five stages from development to production: (1) taking a biopsy of animal cells, (2) cell banking, (3) cell growth, (4) harvesting, and (5) food processing (Fig. 3) (Benson and Greene, 2023; Congressional Research Service, 2023). Regarding safety evaluation, the U.S. Food and Drug Administration (FDA) and the U.S. Department of Agriculture (USDA) jointly announced in February 2019 that they would be responsible. The FDA is responsible for cell collection, cell lines, and differentiation, while the USDA is responsible from the harvesting stage onwards (Benson and Greene, 2023; Congressional Research Service, 2023). This means that the FDA oversees regulation of basic facilities for safe production, including cell line management and differentiation, to comply with Good Manufacturing Practices and preventive control regulations as well as the Federal Food, Drug, and Cosmetic Act (FFDCA; 21 U.S.C. §§301 et seq.). Subsequent USDA oversight ensures compliance with facility design requirements for safe production during the processing of cultured meat into food products. Furthermore, USDA and FDA jointly regulate the production and labeling of cell-cultured meat (Fig. 3).

In 2022, the U.S. FDA approved premarket review for cultured chicken meat from two companies, Good Meat and UPSIDE Foods (Benson and Greene, 2023; Congressional Research Service, 2023). On June 30, 2023, the USDA and FSIS (Food Safety and Inspection Service) directed both companies to label their cultured chicken meat products as “cell-cultivated chicken” in their facilities. In July 2023, Good Meat and UPSIDE Foods sold their cultured chicken meat products to restaurants in Washington, DC, and San Francisco, respectively.

Safety assessment data submitted based on premarket notices for UPSIDE Foods' cultured chicken meat included information on Good Manufacturing Practices (GMP) facilities and Standard Operating Procedures (SOPs) for cultured meat production (Schulze, 2021). Additionally, they provided results for biological, chemical, and physical hazards for risk assessment, which summarized safety evaluation of processed foods. Currently, there is no safety evaluation for raw materials of cultured meat. UPSIDE Foods has argued that foods derived from genetically modified organisms are inherently not harmful, citing federal government regulation (51 FR 23302, 1986; Schulze, 2021). They have also provided evidence that proteins derived from genes used are equivalent to those in regularly consumed foods. Moreover, there are no specific regulations governing the production of cultured meat. Therefore, the following information must be submitted under the GRAS regulations: name of the GRAS material, method of use, detailed safety evaluation (Heo et al., 2024) (Table 1).

### Safety evaluation system in Korea

In South Korea, under the Food Hygiene Act, food ingredients without an established history of consumption can be temporarily designated as novel food ingredients. Although cultured meat originates from animal cells, it is viewed differently between the United States, where it is considered equivalent to traditional animal products, and Europe, where it is regarded as a novel ingredient. In South Korea, cultured meat falls under the category of novel food ingredients (Lee et al., 2022). Novel food ingredients refer to those not listed in the list of food ingredients provided by South Korea based on established consumption history and lack of traditional consumption basis domestically. To be included as a novel food ingredient, documents must be submitted according to the temporarily Standards and Regulations for Food Products by the Ministry of Food and Drug Safety (MFDS) (MFDS, 2024). After the applicant submits the documents, they will undergo a review by the Ministry of Food and Drug Safety, which will then proceed with the review after consulting with a group of experts and deliberation by the Food Sanitation Deliberation Committee (Table 1). Additionally, specific submission requirements are established for food ingredients such as cultured meat utilizing new technologies (MFDS, 2024). In order for cultured meat to be recognized as a temporary food ingredient, information about the cells must be specified in addition to the existing documents for recognition as a temporary food ingredient (Table 1). For safety assessment of cell-cultured food ingredients, toxicological test data and intake evaluation data must be submitted (Lee et al., 2022). Toxicological tests must be conducted by Good Laboratory Practice (GLP) institutions, which may incur high experimental costs.

## New horizon

To date, Singapore, the USA, and Israel have approved the sale of cultured meat. Singapore granted approval in 2020, and the USA followed in 2022, allowing cultured meat to be reviewed and sold in the market. However, when selling cultured meat, terms such as “cell-cultivated chicken,” “cultured,” or “cell-based” must be used to clearly indicate that it is cultured meat. Apart from these three countries, no other nation has yet approved the sale of cultured meat.

Cultured meat is a novel food that has emerged through the application of cell culturing, a new technology. Although cultured meat originates from animals, to claim that they are equivalent to traditional animal-derived foods, there should be evidence such as identical nutritional compositions or equivalence in terms of genome, transcriptome, metabolome, etc. However, so far, there have been no presented results regarding genome, transcriptome, or metabolome equivalence, apart from nutritional components.

Upside Foods provided the FDA with nutritional analysis data in its safety assessment documents for cell-cultured chicken. In the case of cultured meat, the analysis included moisture, protein, fat, ash, and carbohydrate content, following the same order as traditional meat. However, cultured meat showed higher levels of lead, cadmium, etc., than conventional chicken. Although the order of nutritional content is the same, trace content is different. Other results are insufficient. For instance, an extract derived from ginseng root is not the same as ginseng itself. Considering cultured meat cells as meat just because they originate from the same animal is a premature decision. Besides nutritional components, additional equivalence evaluations should be provided. If these results differ from those of traditional meat, it indicates that this is a new food produced through cell culturing. Therefore, safety assessments for this new food should be conducted. Considering one aspect of the safety assessment for cultured meat, it is expected that the direction of cultured meat policies will vary by country due to differences in regulations (Lee et al., 2020). Specifically, the regulations concerning substances used in the production of cultured meat are likely to differ from country to country, and a specialized regulatory system will be required to address these differences (Bhat et al., 2019).
